# Anti-Toxin and Post-Diphtherial Paralysis

**Published:** 1898-09-10

**Authors:** 


### ANTI-TOXIN AND POST-DIPHTHERIAL PARALYSIS.

The relation between the use of anti-toxin and the occurrence of post-diphtherial paralysis is a matter of considerable interest, especially in view of the statement that since the introduction of the anti-toxin treatment the number of such cases has increased. Dr. Goodall says, however, that, although since the introduction of the anti-toxin treatment the number of such cases has certainly increased, the reason probably is that patients now recover, or, at any rate, live long enough to show symptoms of paralysis, who without anti-toxin would have died at an earlier period of the disease. He also adds that, though the number of cases of paralysis has increased, the number of fatal cases has diminished, and that the earlier the anti-toxin treatment is commenced the smaller is the proportion in which paralysis occurs. It is probable that it is not fully recognised how early in the disease this so-called post-diphtherial paralysis may arise. According to the investigations of Dr. Miller, of the South-Eastern Hospital, the bulk of the palatal cases occur between the fifth and the fifteenth day; of the oculo-motor paralyses between the fourth and the seventeenth day: and of the cardiac paralyses between the fifth and the tenth day; and Dr. Sims Woodhead has pointed out that, even during the first 24 hours during which the toxin may be acting on the nerve cells, certain changes are produced in them which, although perhaps temporary, may lead to degeneration of the nerves to which these cells serve as trophic centres. It would appear, in fact, from what he said at Edinburgh, that the poison acts directly and rapidly on the nerve cells even when it is only later that, under the influence of over-work and ill-nutrition, the resulting paralysis becomes unmasked; and that one of the reasons why the heart fails earliest and most frequently is that it is the organ which gets the least rest, while, on the other hand, it is the organ which most depends on the perfection of nervous arrangements. The practical outcome of all this is that anti-toxin should be used early, before degenerative changes have been set up, and that enough anti-toxin should be used to neutralise not only the lethal effects of the diphtheria toxin, but also its local and paralysis-producing action. There seems to be complete unanimity as to this.

